# Supplementary material for: Differences in subcortico-cortical interactions identified from connectome and microcircuit models in autism
Source: Nat Commun. 2021 Apr 13;12:2225. doi: 10.1038/s41467-021-21732-0 (PMC8044226; doi:10.1038/s41467-021-21732-0)
Supplement: Supplementary file 1 — Supplementary Information [file 41467_2021_21732_MOESM1_ESM.pdf]

Supplementary Information

DIFFERENCES IN SUBCORTICO-CORTICAL INTERACTIONS IDENTIFIED FROM CONNECTOME AND MICROCIRCUIT MODELS IN AUTISM

Bo-yong Park[1*], Seok-Jun Hong[1,2,3,4], Sofie L. Valk[5,6], Casey Paquola[1], Oualid Benkarim[1], Richard A. I. Bethlehem[7,8], Adriana Di Martino[2], Michael P. Milham[2], Alessandro Gozzi[9], B. T. Thomas Yeo[10,11,12,13,14], Jonathan Smallwood[15,16], Boris C. Bernhardt[1*]

[1]McConnell Brain Imaging Centre, Montreal Neurological Institute and Hospital, McGill University, Montreal, Quebec, Canada
[2]Center for the Developing Brain, Child Mind Institute, New York City, New York, United States of America
[3]Center for Neuroscience Imaging Research, Institute for Basic Science, Sungkyunkwan University, Suwon, South Korea
[4]Department of Biomedical Engineering, Sungkyunkwan University, Suwon, South Korea
[5]Forschungszentrum Julich, Germany
[6]Max Planck Institute for Cognitive and Brain Sciences, Leipzig, Germany
[7]Autism Research Centre, Department of Psychiatry, University of Cambridge, Cambridge, United Kingdom
[8]Brain Mapping Unit, Department of Psychiatry, University of Cambridge, Cambridge, United Kingdom
[9]Istituto Italiano di Tecnologia, Centre for Neuroscience and Cognitive Systems @ UNITN, Rovereto, Italy
[10]Department of Electrical and Computer Engineering, National University of Singapore, Singapore, Singapore
[11]Centre for Sleep and Cognition (CSC) & Centre for Translational Magnetic Resonance Research (TMR), National University of Singapore, Singapore, Singapore
[12]N.1 Institute for Health & Institute for Digital Medicine (WisDM), National University of Singapore, Singapore, Singapore
[13]Martinos Center for Biomedical Imaging, Massachusetts General Hospital, Charlestown, Massachusetts, United States of America
[14]NUS Graduate School for Integrative Sciences and Engineering, National University of Singapore, Singapore, Singapore
[15]Department of Psychology, York Neuroimaging Centre, University of York, York, United Kingdom
[16]Department of Psychology, Queen's University, Kingston, Ontario, Canada

***Corresponding Authors:**

Bo-yong Park, PhD
Multimodal Imaging and Connectome Analysis Lab
McConnell Brain Imaging Centre
Montreal Neurological Institute and Hospital
McGill University
Montreal, Quebec, Canada
Email: bo.y.park@mcgill.ca

Boris C. Bernhardt, PhD
Multimodal Imaging and Connectome Analysis Lab
McConnell Brain Imaging Centre
Montreal Neurological Institute and Hospital
McGill University
Montreal, Quebec, Canada
Phone: +1-514-398-3579
Email: boris.bernhardt@mcgill.ca

Supplementary Table 1 | Demographic information of the study participants. Means and SDs are reported.

Information		NYU		TCD		P-value
Number (Autism/Control)		29/18		18/19		0.2316*
Age	Autism	9.61 (6.16)	p = 0.8074	14.46 (3.30)	p = 0.2088	0.0037
	Control	10.01 (3.95)		15.83 (3.21)		<0.001
Sex (male:female)	Autism	24:5	p = 0.2432*	18:0	p = 1*	0.0624*
	Control	17:1		19:0		0.2976*
ADOS – Total		10.00 (3.36)		8.72 (2.44)		0.1924
ADOS – Social cognition		7.50 (2.09)		5.78 (2.37)		0.0226
ADOS – Communication		2.5 (1.70)		2.94 (0.87)		0.3260
ADOS – Repeated behavior/interest		1.40 (1.27)		0.22 (0.55)		<0.001

*Chi-squared

Abbreviations: SD, standard deviation; NYU, New York University Langone Medical Center; TCD, Trinity College Dublin; ADOS, Autism Diagnostic Observation Schedule.

Supplementary Table 2 | Seminal model of neural organization that contains four cortical hierarchy levels.

Cortical hierarchy	Mesulam 2000 [1]	Brodmann 1909 [2]
Idiotypic	Striate	17
	Auditory	41, 42
	Somatosensory	3a, 3b, 1, 2
	Motor	4, 6
Unimodal association	Upstream peristriate	18, 19
	Inferotemporal	20, 21, 37
	Superior temporal	22
	Superior parietal lobule	5, anterior 7
	Inferior parietal lobule	anterior 40
	Premotor	anterior 6, posterior 8, 44
Heteromodal association	Prefrontal cortex	9, 10, 45, 46, 47, anterior 11, anterior 12, anterior 8
	Posterior parietal	posterior 7, 39, 40
	Lateral temporal	parts of 21 & 37
	Parahippocampal	parts of 36 & 37
Paralimbic	Orbitofrontal cortex	posterior 11, posterior 12, 13
	Insula	14, 15, 16
	Temporal pole	38
	Parahippocampal	27, 28, 35
	Cingulate	23, 24, 25, 26, 29, 30, 31, 32, 33

Adapted from *Paquola et al., 2019* [3].

Supplementary Table 3 | Associations between structural manifolds and autism symptoms with different regularization parameters (ρ) using five-fold nested cross-validation. We reported the selected brain regions on cortical surface with the frequency of selection across different ADOS scores. For prediction performance, means ± SDs are reported. Significance of the prediction was determined by 1,000 permutation tests by randomly shuffling ADOS scores. ADOS scores available in 38 individuals with autism. Source data are provided as a Source Data file.

ρ	Selected brain regions (0–4)	ADOS-T	ADOS-S	ADOS-C	ADOS-R
**0.1**	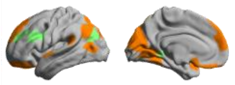	r = 0.29±0.14<br>MAE = 4.75±0.80<br>p = 0.15	r = 0.36±0.06<br>MAE = 1.71±.07<br>p = 0.03	r = 0.49±0.08<br>MAE = 1.29±0.12<br>p = 0.01	r = 0.24±0.07<br>MAE = 0.87±0.03<br>p = 0.16
**0.2**	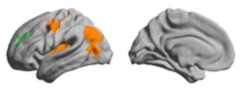	r = 0.43±0.05<br>MAE = 2.13±0.08<br>p = 0.01	r = 0.28±0.06<br>MAE = 1.86±0.05<br>p = 0.11	r = 0.44±0.08<br>MAE = 1.11±0.10<br>p = 0.02	r = 0.26±0.07<br>MAE = 0.86±0.03<br>p = 0.15
**0.3**	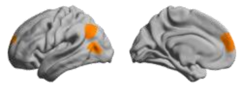	r = 0.32±0.05<br>MAE = 2.14±0.06<br>p = 0.06	r = 0.21±0.09<br>MAE = 1.83±0.07<br>p = 0.25	r = 0.38±0.05<br>MAE = 1.01±0.03<br>p = 0.02	r = 0.24±0.08<br>MAE = 0.86±0.03<br>p = 0.17
**0.4**	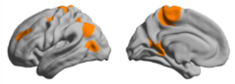	r = 0.51±0.06<br>MAE = 2.03±0.14<br>p < 0.01	r = 0.29±0.05<br>MAE = 1.67±0.05<br>p = 0.09	r = 0.49±0.05<br>MAE = 0.93±0.04<br>p < 0.01	r = 0.19±0.07<br>MAE = 0.87±0.04<br>p = 0.28
**0.5**	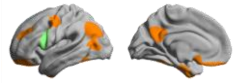	r = 0.33±0.09<br>MAE = 2.38±0.16<br>p = 0.08	r = 0.28±0.06<br>MAE = 1.77±0.05<br>p = 0.11	r = 0.53±0.06<br>MAE = 0.99±0.09<br>p < 0.01	r = 0.25±0.07<br>MAE = 0.86±0.03<br>p = 0.16
**0.6**	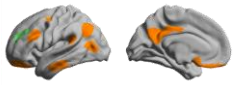	r = 0.47±0.06<br>MAE = 2.07±0.12<br>p = 0.01	r = 0.43±0.07<br>MAE = 1.79±0.09<br>p = 0.01	r = 0.57±0.03<br>MAE = 0.89±0.03<br>p < 0.01	r = 0.33±0.09<br>MAE = 0.89±0.05<br>p = 0.09
**0.7**	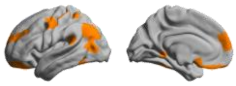	r = 0.37±0.07<br>MAE = 2.31±0.13<br>p = 0.04	r = 0.38±0.07<br>MAE = 1.80±0.08<br>p = 0.04	r = 0.62±0.06<br>MAE = 1.02±0.09<br>p < 0.01	r = 0.25±0.07<br>MAE = 0.86±0.03<br>p = 0.16
**0.8**	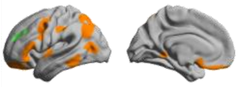	r = 0.48±0.07<br>MAE = 2.24±0.19<br>p = 0.01	r = 0.36±0.08<br>MAE = 1.91±0.14<br>p = 0.06	r = 0.47±0.06<br>MAE = 0.90±0.05<br>p = 0.01	r = 0.24±0.08<br>MAE = 0.86±0.03<br>p = 0.18
**0.9**	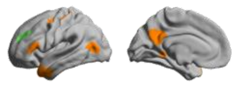	r = 0.43±0.06<br>MAE = 2.17±0.11<br>p = 0.01	r = 0.52±0.08<br>MAE = 1.73±0.11<br>p = 0.01	r = 0.35±0.06<br>MAE = 1.00±0.05<br>p = 0.04	r = 0.27±0.07<br>MAE = 0.85±0.03<br>p = 0.13
**1.0**	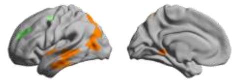	r = 0.53±0.05<br>MAE = 2.17±0.15<br>p < 0.01	r = 0.51±0.06<br>MAE = 1.62±0.09<br>p < 0.01	r = 0.21±0.08<br>MAE = 1.12±0.04<br>p = 0.27	r = 0.31±0.08<br>MAE = 0.94±0.07<br>p = 0.09

Abbreviations: SD, standard deviation; ADOS, Autism Diagnostic Observation Schedule; T, total; S, social cognition; C, communication; R, repeated behavior/interest; MAE, mean absolute error.

Supplementary Table 4 | Associations between structural manifolds and autism symptoms with different regularization parameters (*ρ*) using three-fold nested cross-validation. We reported the selected brain regions on cortical surface with the frequency of selection across different ADOS scores. For prediction performance, means ± SDs are reported. Significance of the prediction was determined by 1,000 permutation tests by randomly shuffling ADOS scores. ADOS scores available in 38 individuals with autism. Source data are provided as a Source Data file.

ρ	Selected brain regions (0–4)	ADOS-T	ADOS-S	ADOS-C	ADOS-R
**0.1**	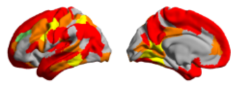	r = 0.28±0.12<br>MAE = 2.36±0.16<br>p = 0.14	r = 0.02±0.14<br>MAE = 3.81±0.38<br>p = 0.56	r = 0.27±0.14<br>MAE = 1.87±.21<br>p = 0.19	r = 0.23±0.12<br>MAE = 0.91±0.06<br>p = 0.22
**0.2**	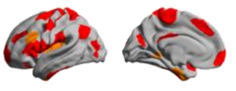	r = 0.15±0.14<br>MAE = 2.39±0.26<br>p = 0.38	r = 0.29±0.08<br>MAE = 1.69±0.08<br>p = 0.11	r = 0.18±0.15<br>MAE = 1.91±0.19<br>p = 0.35	r = 0.21±0.09<br>MAE = 0.84±0.04<br>p = 0.25
**0.3**	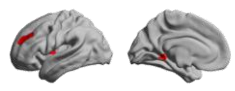	r = 0.43±0.06<br>MAE = 2.14±0.09<br>p = 0.01	r = 0.29±0.07<br>MAE = 1.68±0.08<br>p = 0.10	r = 0.34±0.08<br>MAE = 1.02±0.05<br>p = 0.07	r = 0.23±0.10<br>MAE = 0.91±0.06<br>p = 0.22
**0.4**	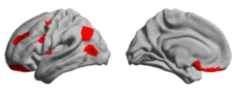	r = 0.37±0.10<br>MAE = 2.27±0.18<br>p = 0.07	r = 0.28±.07<br>MAE = 1.87±0.08<br>p = 0.12	r = 0.44±0.08<br>MAE = 0.95±.05<br>p = 0.01	r = 0.26±0.09<br>MAE = 0.87±0.04<br>p = 0.17
**0.5**	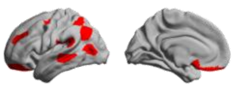	r = 0.15±0.11<br>MAE = 2.44±0.11<br>p = 0.38	r = 0.33±0.12<br>MAE = 1.98±0.19<br>p = 0.11	r = 0.38±0.07<br>MAE = 1.01±0.04<br>p = 0.03	r = 0.24±0.11<br>MAE = 0.87±0.05<br>p = 0.21
**0.6**	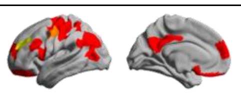	r = 0.40±0.10<br>MAE = 2.28±0.18<br>p = 0.04	r = 0.20±0.13<br>MAE = 2.80±0.33<br>p = 0.27	r = 0.20±0.19<br>MAE = 2.87±0.92<br>p = 0.27	r = 0.21±0.12<br>MAE = 0.90±.06<br>p = 0.28
**0.7**	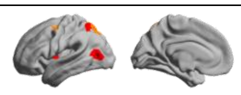	r = 0.40±.07<br>MAE = 2.18±0.14<br>p = 0.03	r = 0.37±0.08<br>MAE = 1.80±0.10<br>p = 0.05	r = 0.34±0.09<br>MAE = 1.01±0.05<br>p = 0.07	r = 0.24±0.11<br>MAE = 0.87±0.05<br>p = 0.20
**0.8**	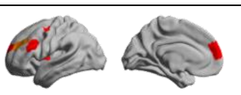	r = 0.42±0.08<br>MAE = 2.18±0.12<br>p = 0.02	r = 0.39±0.08<br>MAE = 1.78±0.10<br>p = 0.03	r = 0.49±0.06<br>MAE = 0.93±0.06<br>p < 0.01	r = 0.18±0.10<br>MAE = 0.85±0.05<br>p = 0.31
**0.9**	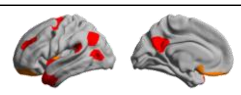	r = 0.36±0.10<br>MAE = 2.41±0.15<br>p = 0.07	r = 0.35±0.13<br>MAE = 2.13±0.24<br>p = 0.09	r = 0.44±0.08<br>MAE = 0.96±0.06<br>p = 0.02	r = 0.22±0.13<br>MAE = 0.96±0.09<br>p = 0.28
**1.0**	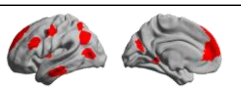	r = 0.28±0.10<br>MAE = 2.39±0.15<br>p = 0.15	r = 0.18±0.13<br>MAE = 1.90±0.16<br>p = 0.35	r = 0.36±0.13<br>MAE = 1.12±0.13<br>p = 0.09	r = 0.27±0.09<br>MAE = 0.86±0.05<br>p = 0.15

Abbreviations: SD, standard deviation; ADOS, Autism Diagnostic Observation Schedule; T, total; S, social cognition; C, communication; R, repeated behavior/interest; MAE, mean absolute error.

Supplementary Table 5 | Associations between structural manifolds and autism symptoms with different regularization parameters (*ρ*) using five-fold nested cross-validation for dataset from NYU site. We reported the selected brain regions on cortical surface with the frequency of selection across different ADOS scores. For prediction performance, means ± SDs are reported. Significance of the prediction was determined by 1,000 permutation tests by randomly shuffling ADOS scores. ADOS scores available in 20 individuals with autism. Source data are provided as a Source Data file.

ρ	Selected brain regions 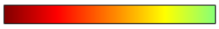 0 4	ADOS-T	ADOS-S	ADOS-C	ADOS-R
0.1	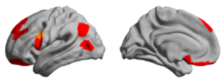	r = ±0.45±0.06<br>MAE = 2.52±0.15<br>p = 0.05	r = 0.53±0.08<br>MAE = 1.36±0.10<br>p=< 0.03	r = 0.43±0.09<br>MAE = 1.32±0.12<br>p = 0.08	r = 0.68±0.05<br>MAE = 0.79±0.06<br>p = 0.01
0.2	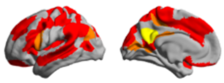	r = 0.47±0.13<br>MAE = 4.59±0.59<br>p = 0.08	r = 0.50±0.11<br>MAE = 1.38±0.10<br>p = 0.05	r = 0.27±0.23<br>MAE = 2.46±1.53<br>p = 0.30	r = 0.62±0.06<br>MAE = 0.86±0.07<br>p = 0.01
0.3	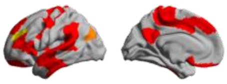	r = 0.66±0.11<br>MAE = 4.15±0.67<br>p = 0.01	r = 0.51±0.10<br>MAE = 1.36±0.10<br>p = 0.04	r = 0.30±0.24<br>MAE = 2.68±2.77<br>p = 0.29	r = 0.36±0.11<br>MAE = 0.95±.08<br>p = 0.15
0.4	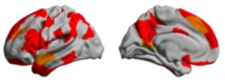	r = 0.51±0.11<br>MAE = 4.33±0.55<br>p = 0.05	r = 0.51±0.17<br>MAE = 1.49±0.20<br>p = 0.07	r = 0.29±0.18<br>MAE = 2.16±0.93<br>p = 0.27	r = 0.69±0.12<br>MAE = 0.82±0.15<br>p = 0.01
0.5	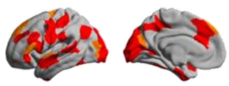	r = 0.38±0.20<br>MAE = 8.17±2.48<br>p = 0.19	r = 0.30±0.19<br>MAE = 4.90±3.66<br>p = 0.26	r = 0.67±0.08<br>MAE = 1.06±0.14<br>p < 0.01	r = 0.82±0.02<br>MAE = 0.59±0.05<br>p < 0.01
0.6	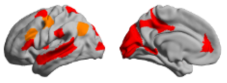	r = 0.34±0.09<br>MAE = 2.46±0.22<br>p = 0.17	r = 0.51±0.12<br>MAE = 1.68±0.27<br>p = 0.05	r = 0.38±0.25<br>MAE = 2.52±4.74<br>p = 0.20	r = 0.60±0.06<br>MAE = 0.86±0.05<br>p = 0.01
0.7	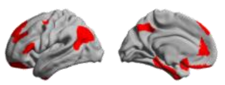	r = 0.59±0.10<br>MAE = 3.04±0.54<br>p = 0.02	r = 0.76±0.04<br>MAE = 1.13±0.10<br>p < 0.01	r = 0.37±0.09<br>MAE = 1.35±0.09<br>p = 0.13	r = 0.83±0.02<br>MAE = 0.61±0.05<br>p < 0.01
0.8	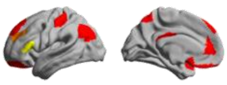	r = 0.09±0.23<br>MAE = 31.2±28.8<br>p = 0.5	r = 0.78±0.04<br>MAE = 1.04±0.10<br>p < 0.01	r = 0.74±0.03<br>MAE = 0.92±0.08<br>p < 0.01	r = 0.74±0.08<br>MAE = 0.82±0.12<br>p < 0.01
0.9	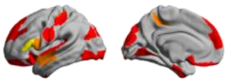	r = 0.23±0.23<br>MAE = 8.78±4.38<br>p = 0.38	r = 0.47±0.14<br>MAE = 2.61±0.76<br>p = 0.09	r = 0.52±0.08<br>MAE = 1.25±0.14<br>p = 0.03	r = 0.81±0.04<br>MAE = 0.58±0.06<br>p < 0.01
1.0	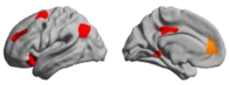	r = 0.61±0.08<br>MAE = 1.97±0.22<br>p = 0.01	r = 0.32±0.09<br>MAE = 1.56±0.13<br>p = 0.18	r = 0.49±0.10<br>MAE = 1.18±0.13<br>p = 0.05	r = 0.62±0.05<br>MAE = 0.85±0.04<br>p = 0.01

Abbreviations: SD, standard deviation; NYU, New York University Langone Medical Center; ADOS, Autism Diagnostic Observation Schedule; T, total; S, social cognition; C, communication; R, repeated behavior/interest; MAE, mean absolute error.

Supplementary Table 6 | Associations between structural manifolds and autism symptoms with different regularization parameters (*ρ*) using five-fold nested cross-validation for dataset from TCD site. We reported the selected brain regions on cortical surface with the frequency of selection across different ADOS scores. For prediction performance, means ± SDs are reported. Significance of the prediction was determined by 1,000 permutation tests by randomly shuffling ADOS scores. ADOS scores available in 18 individuals with autism. Source data are provided as a Source Data file.

ρ	**Selected brain regions** 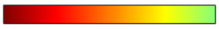	**ADOS-T**	**ADOS-S**	**ADOS-C**	**ADOS-R**
**0.1**	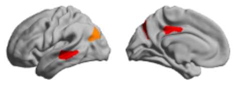	r = 0.32±0.08<br>MAE = 1.65±0.10<br>p = 0.23	r = 0.26±0.07<br>MAE = 1.66±0.12<br>p = 0.32	r = 0.56±0.11<br>MAE = 0.65±0.11<br>p = 0.04	r = 0.43±0.16<br>MAE = 0.37±0.03<br>p = 0.12
**0.2**	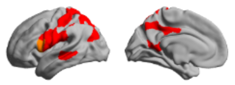	r = 0.43±0.07<br>MAE = 1.60±0.11<br>p = 0.08	r = 0.18±0.11<br>MAE = 2.54±0.43<br>p = 0.43	r = 0.13±0.14<br>MAE = 1.74±0.38<br>p = 0.55	r = 0.46±0.14<br>MAE = 0.35±0.04<br>p = 0.10
**0.3**	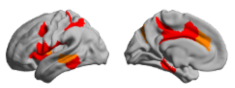	r = 0.47±0.10<br>MAE = 1.75±0.22<br>p = 0.06	r = 0.17±0.12<br>MAE = 4.77±1.11<br>p = 0.49	r = 0.62±0.10<br>MAE = 0.61±0.11<br>p = 0.02	r = 0.26±0.19<br>MAE = 0.39±0.04<br>p = 0.33
**0.4**	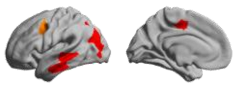	r = 0.59±0.06<br>MAE = 1.44±0.11<br>p = 0.01	r = 0.36±0.08<br>MAE = 1.75±0.11<br>p = 0.17	r = 0.59±0.05<br>MAE = 0.56±0.03<br>p = 0.01	r = 0.53±0.17<br>MAE = 0.43±0.06<br>p = 0.06
**0.5**	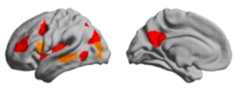	r = 0.26±0.11<br>MAE = 4.09±0.61<br>p = 0.33	r = 0.09±0.09<br>MAE = 1.66±0.10<br>p = 0.67	r = 0.59±0.04<br>MAE = 0.56±0.03<br>p = 0.01	r = 0.07±0.16<br>MAE = 3.84±3.48<br>p = 0.57
**0.6**	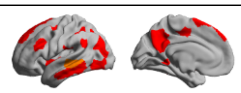	r = 0.33±0.08<br>MAE = 1.99±0.17<br>p = 0.16	r = 0.27±0.14<br>MAE = 4.61±1.20<br>p = 0.33	r = 0.31±0.08<br>MAE = 0.66±0.05<br>p = 0.24	r = 0.08±0.18<br>MAE = 1.39±0.65<br>p = 0.55
**0.7**	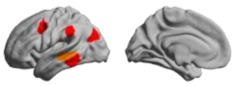	r = 0.15±0.11<br>MAE = 1.86±0.10<br>p = 0.56	r = 0.50±0.06<br>MAE = 1.68±0.14<br>p = 0.03	r = 0.59±0.05<br>MAE = 0.56±0.04<br>p = 0.01	r = 0.41±0.18<br>MAE = 0.39±0.06<br>p = 0.17
**0.8**	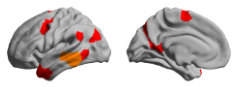	r = 0.31±0.16<br>MAE = 4.89±1.75<br>p = 0.26	r = 0.30±0.06<br>MAE = 1.62±0.08<br>p = 0.22	r = 0.67±0.04<br>MAE = 0.53±0.03<br>p < 0.01	r = 0.43±0.14<br>MAE = 0.37±0.04<br>p = 0.14
**0.9**	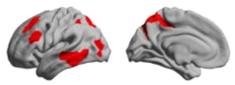	r = 0.40±0.04<br>MAE = 1.68±0.23<br>p = 0.05	r = 0.39±0.11<br>MAE = 1.89±0.21<br>p = 0.16	r = 0.60±0.08<br>MAE = 0.58±0.08<br>p = 0.02	r = 0.28±0.15<br>MAE = 0.39±0.04<br>p = 0.33
**1.0**	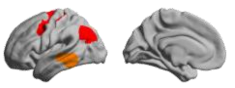	r = 0.49±0.07<br>MAE = 1.76±0.17<br>p = 0.03	r = 0.32±0.14<br>MAE = 2.47±0.41<br>p = 0.25	r = 0.57±0.08<br>MAE = 0.62±0.09<br>p = 0.02	r = 0.41±0.15<br>MAE = 0.37±0.03<br>p = 0.13

Abbreviations: SD, standard deviation; TCD, Trinity College Dublin; ADOS, Autism Diagnostic Observation Schedule; T, total; S, social cognition; C, communication; R, repeated behavior/interest; MAE, mean absolute error.

Supplementary Table 7 | Associations between streamline cross-section and autism symptoms with different regularization parameters (ρ) using five-fold nested cross-validation. We charted the selected connections with circular plots reordered based on established functional communities [4]. The line width represents the frequency of selection across different ADOS scores. The location of the selected connections is represented on the cortical surface. For prediction performance, means ± SDs are reported. Significance of the prediction was determined by 1,000 permutation tests by randomly shuffling ADOS scores. ADOS scores available in 38 individuals with autism. Source data are provided as a Source Data file.

ρ	Selected connections and brain regions 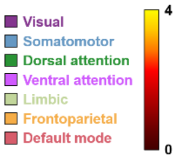	ADOS-T	ADOS-S	ADOS-C	ADOS-R
**0.1**	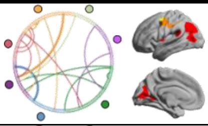	r = 0.74±0.03<br>MAE = 1.53±0.09<br>p < 0.01	r = 0.32±0.07<br>MAE = 1.90±0.06<br>p = 0.08	r = 0.66±0.04<br>MAE = 0.88±0.09<br>p < 0.01	r = 0.20±0.07<br>MAE = 0.84±0.04<br>p = 0.28
**0.2**	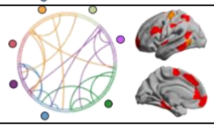	r = 0.52±0.03<br>MAE = 1.99±0.06<br>p < 0.01	r = 0.06±0.16<br>MAE = 21.1±34.3<br>p = 0.50	r = 0.55±0.03<br>MAE = 0.89±0.03<br>p < 0.01	r = 0.32±0.16<br>MAE = 0.89±0.05<br>p = 0.14
**0.3**	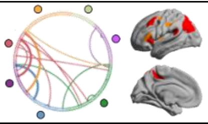	r = 0.63±0.04<br>MAE = 1.83±0.09<br>p < 0.01	r = 0.21±0.06<br>MAE = 2.52±0.26<br>p = 0.22	r = 0.77±0.03<br>MAE = 0.70±0.06<br>p < 0.01	r = 0.01±0.07<br>MAE = 1.10±0.24<br>p = 0.72
**0.4**	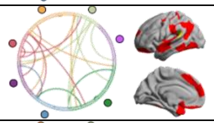	r = 0.52±0.03<br>MAE = 1.99±0.05<br>p < 0.01	r = 0.14±0.09<br>MAE = 2.09±0.23<br>p = 0.43	r = 0.78±0.07<br>MAE = 0.81±0.13<br>p < 0.01	r = 0.06±0.06<br>MAE = 0.97±0.15<br>p = 0.65
**0.5**	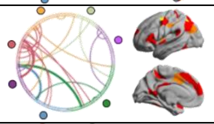	r = 0.65±0.03<br>MAE = 1.93±0.14<br>p < 0.01	r = 0.30±0.11<br>MAE = 1.85±0.07<br>p = 0.16	r = 0.60±0.09<br>MAE = 0.94±0.11<br>p < 0.01	r = 0.17±0.03<br>MAE = 1.15±0.09<br>p = 0.15
**0.6**	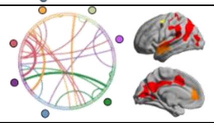	r = 0.73±0.06<br>MAE = 1.79±0.19<br>p < 0.01	r = 0.42±0.06<br>MAE = 1.84±0.17<br>p = 0.03	r = 0.74±0.03<br>MAE = 0.77±0.05<br>p < 0.01	r = 0.14±0.17<br>MAE = 1.04±0.12<br>p = 0.37
**0.7**	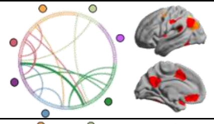	r = 0.59±.06<br>MAE = 1.91±0.15<br>p < 0.01	r = 0.40±0.05<br>MAE = 1.69±0.06<br>p = 0.04	r = 0.81±0.03<br>MAE = 0.60±0.04<br>p < 0.01	r = 0.21±0.32<br>MAE = 0.88±0.08<br>p = 0.11
**0.8**	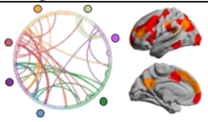	r = 0.31±0.10<br>MAE = 3.80±0.60<br>p = 0.10	r = 0.14±0.11<br>MAE = 3.06±0.41<br>p = 0.44	r = 0.75±0.03<br>MAE = 0.75±0.05<br>p < 0.01	r = 0.19±0.10<br>MAE = 1.21±0.32<br>p = 0.34
**0.9**	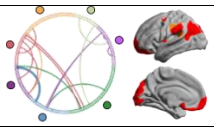	r = 0.57±0.04<br>MAE = 1.85±0.10<br>p < 0.01	r = 0.33±0.12<br>MAE = 1.84±0.09<br>p = 0.10	r = 0.71±0.06<br>MAE = 0.80±0.31<br>p < 0.01	r = 0.07±0.19<br>MAE = 0.93±0.05<br>p = 0.49
**1.0**	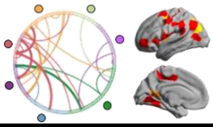	r = 0.63±0.05<br>MAE = 1.87±0.15<br>p < 0.01	r = 0.43±0.09<br>MAE = 2.01±0.21<br>p = 0.02	r = 0.81±0.02<br>MAE = 0.62±0.04<br>p < 0.01	r = 0.26±0.09<br>MAE = 0.90±0.03<br>p = 0.18

Abbreviations: SD, standard deviation; ADOS, Autism Diagnostic Observation Schedule; T, total; S, social cognition; C, communication; R, repeated behavior/interest; MAE, mean absolute error.

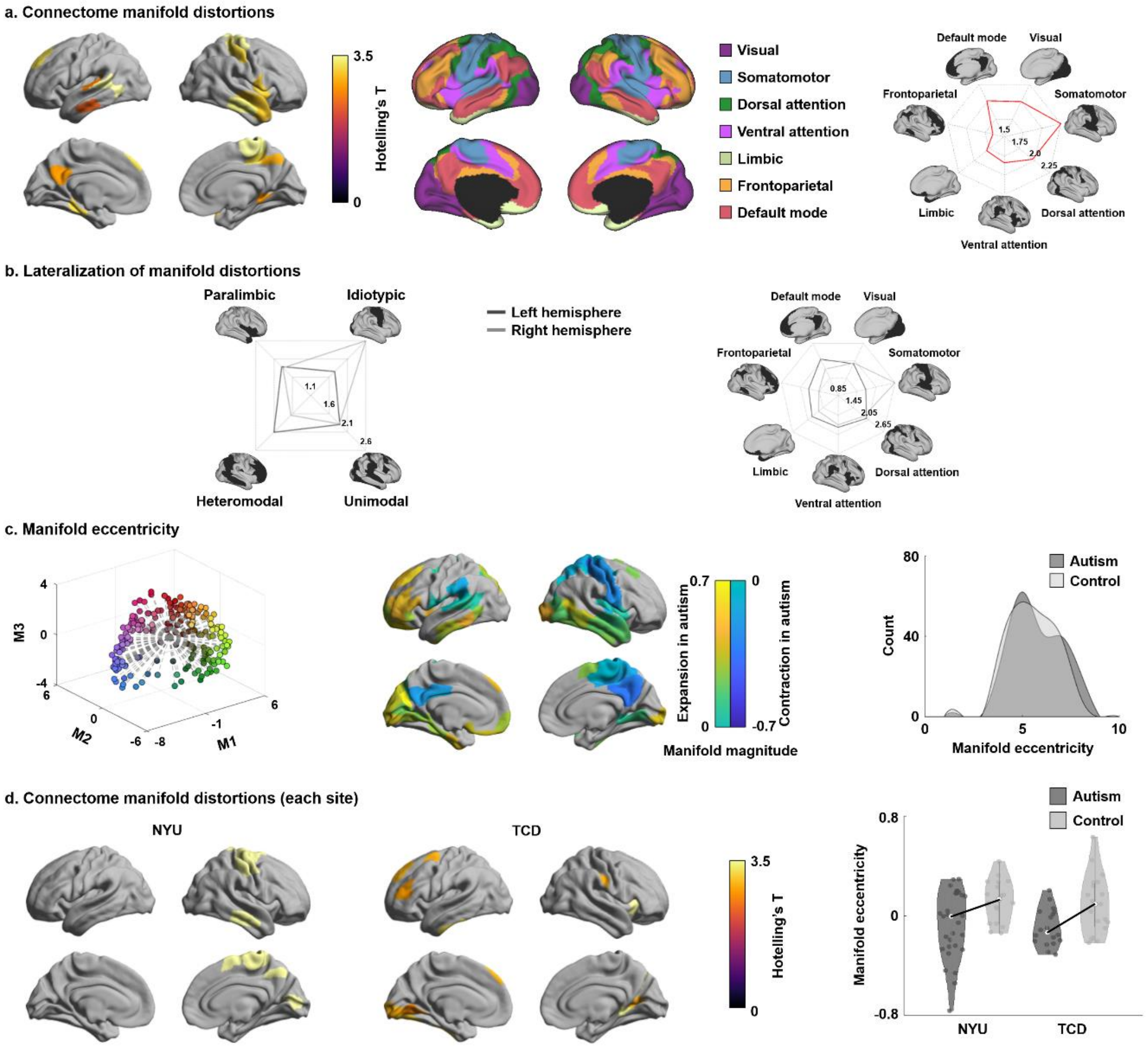

Supplementary Fig. 1 | Connectome manifold distortions. **(a)** The t-statistics of the identified regions that showed significant between-group differences in manifolds between individuals with autism and controls (left). Findings were corrected for multiple comparisons at false discovery rate (FDR) < 0.05. Functional network-wise (middle) [4] summary of the t-statistic values are shown in the radar plot (right). **(b)** Stratification of between-group difference effects for each hemisphere along cortical hierarchical levels (left) [1] and functional networks (right) [4] are presented in the radar plots. **(c)** Manifold eccentricity measured as the Euclidean distance between the template center and each data point (left) and its difference between the groups (middle). The histogram of the manifold eccentricity for each group is reported (right). **(d)** The t-statistics derived from the multivariate group comparison for each site (left and middle). Manifold eccentricity perturbations in individuals with autism and controls of the identified regions for each site are reported on the right. Source data are provided as a Source Data file. Abbreviations: NYU, New York University Langone Medical Center; TCD, Trinity College Dublin.

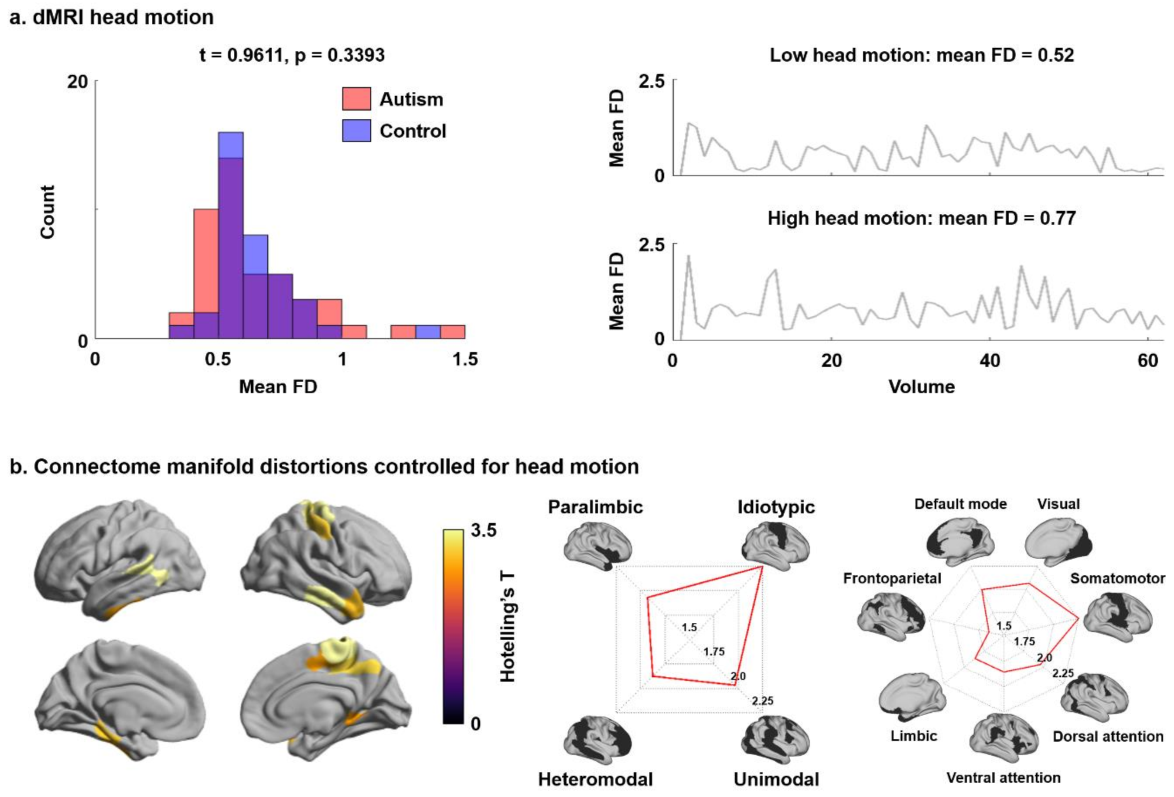

Supplementary Fig. 2 | Head motion effects. (a) Mean framewise displacement (FD) of each group (left) and that of two representative participants with low and high head motion (right). **(b)** The t-statistics derived from the multivariate group comparison between individuals with autism and controls using the three structural manifolds controlled for head motion (left). Stratification of the effects along cortical hierarchical levels (middle) [1] and functional networks (right) [4] are presented in the radar plots. Source data are provided as a Source Data file.

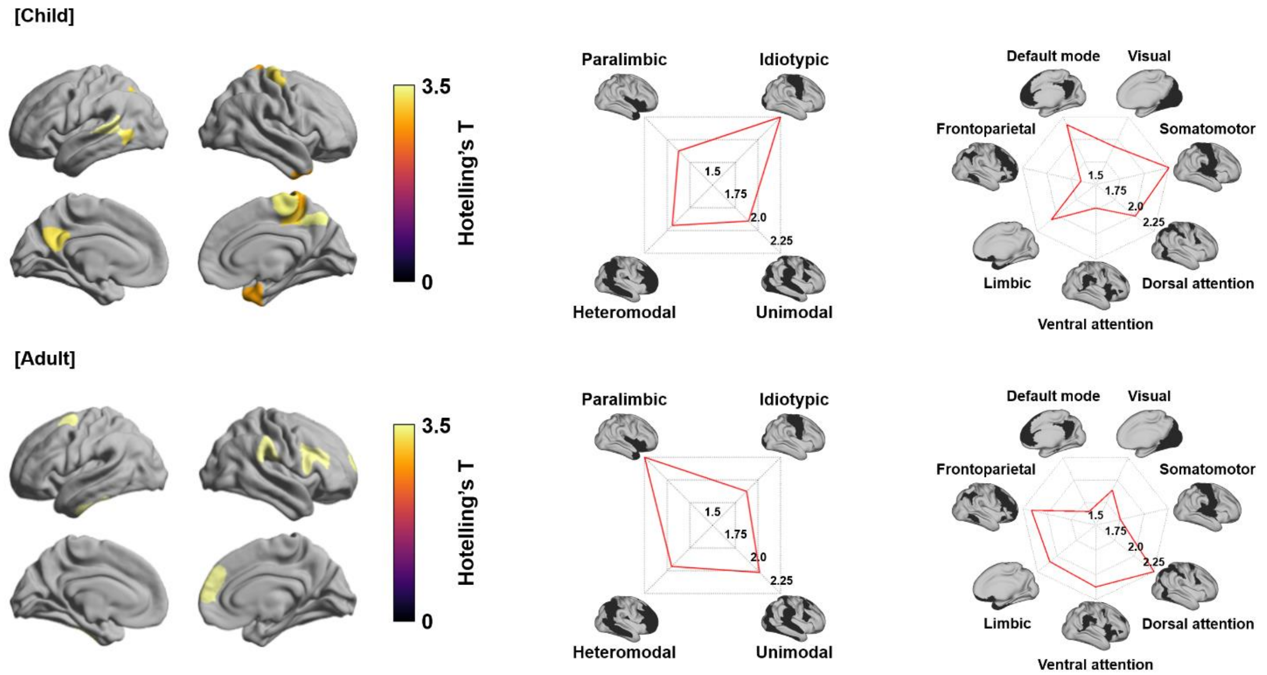

Supplementary Fig. 3 | Age effects. The t-statistics derived from the multivariate group comparison between individuals with autism and controls using the three structural manifolds within children and adult cohorts separately (left). Stratification of the effects along cortical hierarchical levels (middle) [1] and functional networks (right) [4] are presented in the radar plots. Source data are provided as a Source Data file.

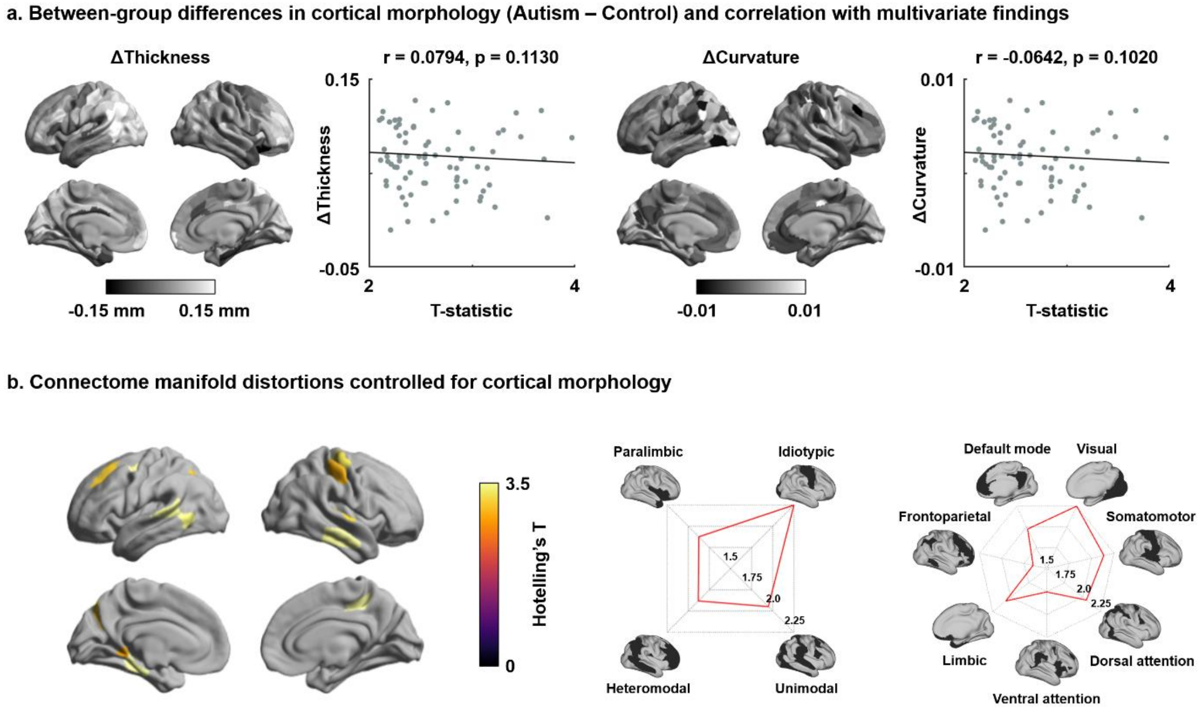

Supplementary Fig. 4 | Morphological associations. (a) Correlation between differences in cortical morphology (i.e., thickness (left) and folding (right)) between individuals with autism and controls and multivariate change pattern. **(b)** The t-statistics derived from the multivariate group comparison between individuals with autism and controls using the three structural manifolds controlled for cortical morphology (left). Stratification of the effects along cortical hierarchical levels (middle) [1] and functional networks (right) [4] are presented in the radar plots. Source data are provided as a Source Data file.

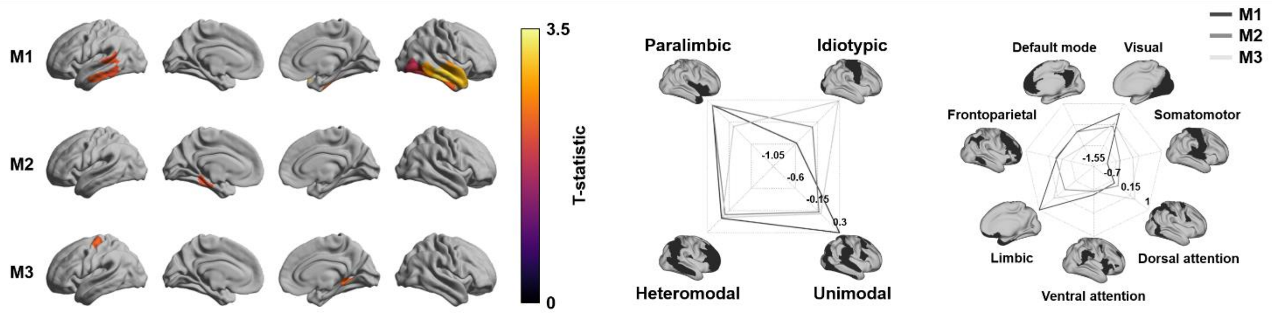

Supplementary Fig. 5 | Distortions in each structural connectome manifold. The t-statistics of the identified regions that showed significant between-group differences in each dimension between individuals with autism and controls (left). Stratification of between-group difference effects along cortical hierarchical levels (middle) [1] and functional networks (right) [4] are presented in the radar plots. Source data are provided as a Source Data file.

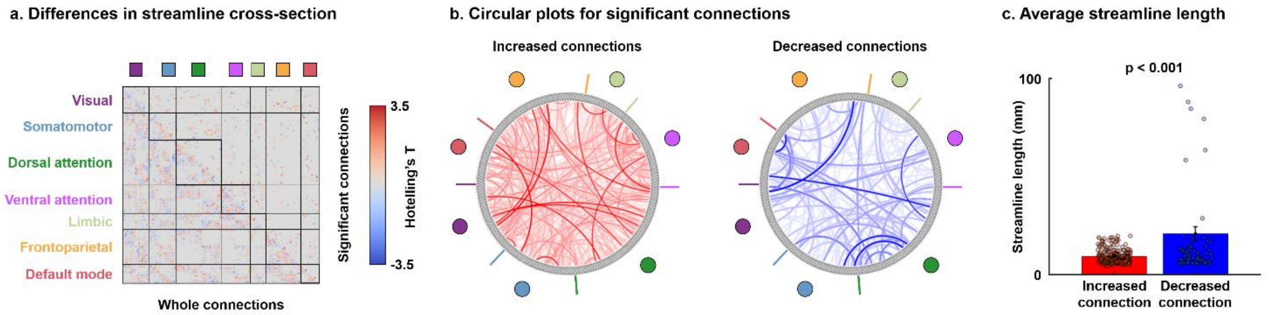

Supplementary Fig. 6 | Edge-wise differences in streamline cross-section between individuals with autism and controls. (a) The t-statistics of the whole connections (lower triangular) showed significant between-group differences (upper triangular) in streamline cross-section between individuals with autism and controls. Findings have been corrected for multiple comparisons at false discovery rate (FDR) < 0.05. **(b)** Circular plots represent the connections that showed significant between-group differences (1,000 permutation tests followed by FDR). Increased/decreased streamline cross-sections in individuals with autism are represented in red/blue. All significant connections are reported with high transparency, and top 50/25/10% t-statistic values are reported with less transparency for better visualization. The solid lines indicate connections with top 5% t-statistic values. **(c)** Average streamline length for top 5% increased (n = 213) and decreased (n = 90) connections. Error bars indicate standard error of the mean. Differences in streamline length between increased and decrease connections were determined using two-sample t-test. Source data are provided as a Source Data file.

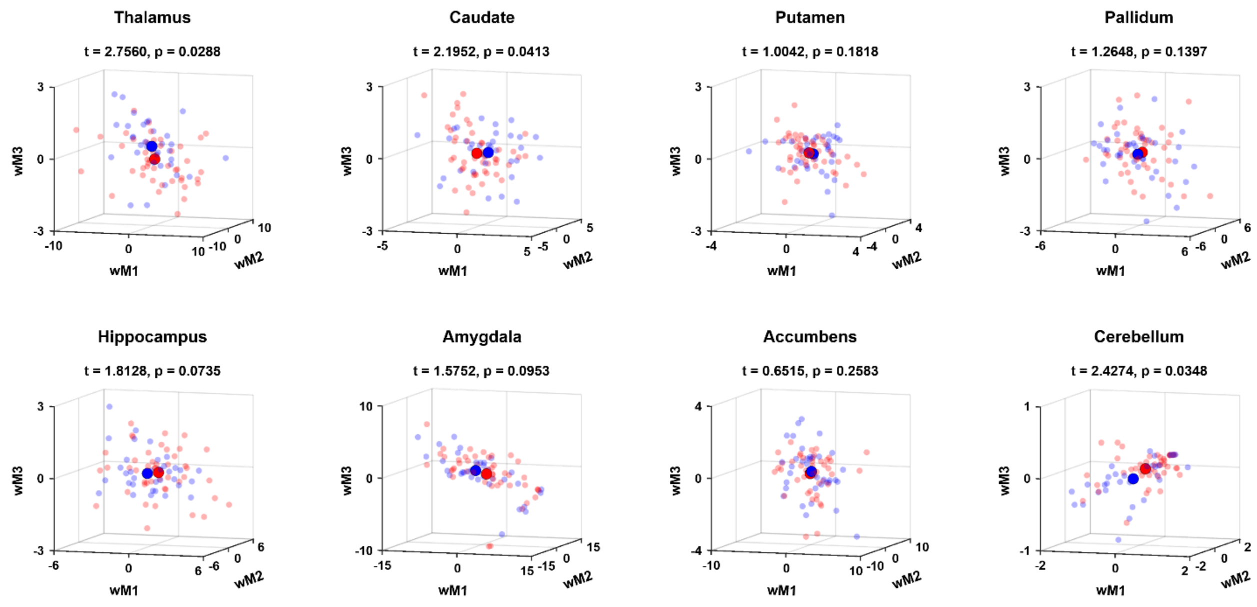

Supplementary Fig. 7 | Subcortical and cerebellar manifold distortions in autism. Cortical manifolds weighted by streamline cross-section of individuals with autism (red) and controls (blue) for each subcortical region and cerebellum. Each transparent dot represents each individual and solid dot indicate the center of the weighted manifolds of each group. The t-statistics of the multivariate between-group comparison in weighted manifolds (wM1, wM2, and wM3) and significance corrected for multiple comparisons at false discovery rate (FDR) < 0.05 are reported. Source data are provided as a Source Data file.

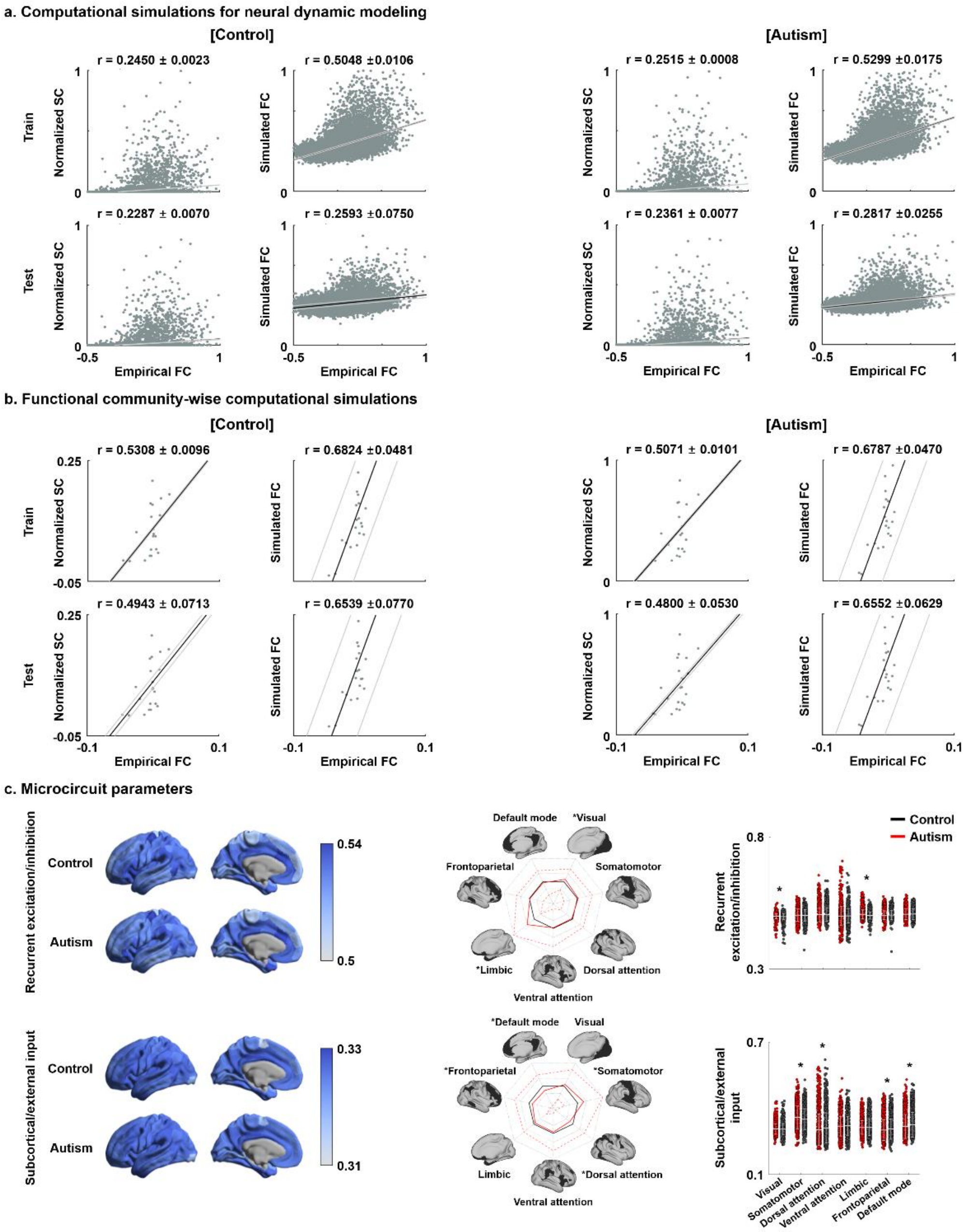

Supplementary Fig. 8 | Microcircuit parameters and biophysical simulations. (a) Linear correlations between empirical functional connectivity (FC) and structural connectivity (SC), and empirical and simulated FC for controls (left) and individuals with autism (right) based on Schaefer atlas with 200 parcels [5] and **(b)** functional communities [4]. Black lines indicate mean correlation and gray lines represent 95% confidence interval across 1,000 bootstrapping. **(c)** Microcircuit parameters of controls and individuals with autism (left). The functional community-wise [4] stratifications of the parameters are presented with the radar plots (middle). Black lines indicate controls normalized to mean zero and standard deviation of one, and red lines indicate individuals with autism normalized according to controls. Solid and dash lines represent mean and standard deviation of the parameters across 1,000 bootstraps, respectively. The scatter plots represent microcircuit parameters across 1,000 bootstraps (right). Each dot is the estimated parameter for each bootstrap. The white horizontal lines indicate mean of the parameter across bootstraps and gray vertical lines are standard deviation. Brain networks with significant between-group differences are represented with asterisks. Source data are provided as a Source Data file.

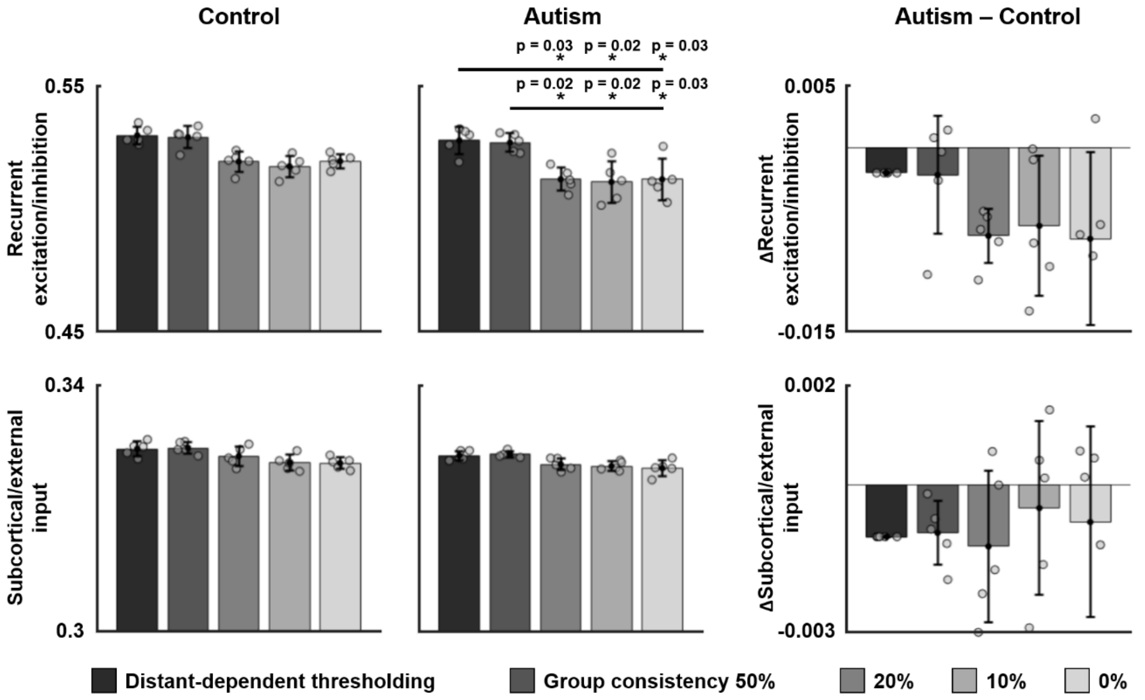

Supplementary Fig. 9 | Microcircuit parameters estimated using different connectome thresholds. The recurrent excitation/inhibition and subcortical/external input estimated from group representative structural connectome matrix with distant-dependent thresholding [6] and consistency of 50, 20, 10, and 0% threshold [7] of each group, as well as between-group differences, across five-fold cross-validations are reported. Each dot represents data from a single cross-validation. Differences in parameters across different thresholds were determined using two-sample t-test, and the significant differences are reported with asterisks. Error bars indicate standard deviation of the parameters across five-fold cross-validations. Source data are provided as a Source Data file.

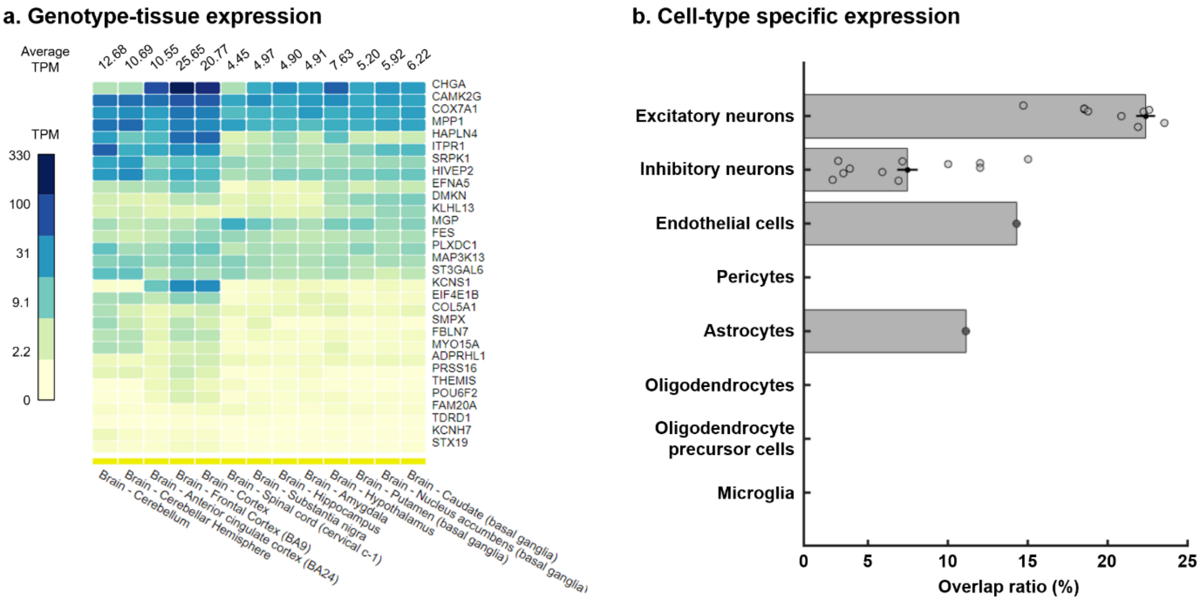

Supplementary Fig. 10 | Gene enrichment using Genotype-Tissue Expression (GTEx) and cell-type specific expression analysis (CSEA). (a) Top 30 ranked genes derived from Neurovault were fed into the multi-gene query of GTEx, and transcripts per million (TPM) of each gene was calculated for different brain structures. Each row represents genes and column represents brain structures. The average TPM value across genes for each brain structure is reported on the top. **(b)** The overlap ratio between the genes expressed for manifold changes and each cell-type specific genes. Error bars represent standard error of the mean for sub-cell-types. Only excitatory (n = 13) and inhibitory (n = 11) neurons have sub-cell-types. Source data are provided as a Source Data file. Abbreviation: BA, Brodmann area.

SUPPLEMENTARY REFERENCES

1. Mesulam, M. M. From sensation to cognition. *Brain* **121**, 1013–1052 (1998).

2. Brodmann, K. *Vergleichende Lokalisationslehre der Grosshirnrinde in ihrenPrinzipien dargestellt auf Grund des Zellenbaues*. (Leipzig : Barth, 1909).

3. Paquola, C. *et al.* Microstructural and functional gradients are increasingly dissociated in transmodal cortices. *PLoS Biol.* **17**, e3000284 (2019).

4. Yeo, B. T. T. *et al.* The organization of the human cerebral cortex estimated by intrinsic functional connectivity. *J. Neurophysiol.* **106**, 1125–1165 (2011).

5. Schaefer, A. *et al.* Local-Global Parcellation of the Human Cerebral Cortex from Intrinsic Functional Connectivity MRI. *Cereb. Cortex* **28**, 3095–3114 (2018).

6. Betzel, R. F., Griffa, A., Hagmann, P. & Miši´c, B. Distance-dependent consensus thresholds for generating group-representative structural brain networks. *Netw. Neurosci.* **3**, 475–496 (2019).

7. Wang, P. *et al.* Inversion of a large-scale circuit model reveals a cortical hierarchy in the dynamic resting human brain. *Sci. Adv.* **5**, eaat7854 (2019).
