# Supplementary material for: Differences in subcortico-cortical interactions identified from connectome and microcircuit models in autism
Source: Nat Commun. 2021 Apr 13;12:2225. doi: 10.1038/s41467-021-21732-0 (PMC8044226; doi:10.1038/s41467-021-21732-0)
Supplement: Supplementary file 3 — Description of Additional Supplementary Files [file 41467_2021_21732_MOESM3_ESM.pdf]

Description of Additional Supplementary Files

File Name: Supplementary Data 1.

Description: Significant gene lists correlated with multivariate change pattern. Gene symbol with name and t-statistic, as well as false discovery rate (FDR) corrected p-value, are reported in the Supplementary Data file (Supplementary Data1.xlsx).
